# Supplementary material for: Exploring the Mycovirus Universe: Identification, Diversity, and Biotechnological Applications
Source: J Fungi (Basel). 2023 Mar 15;9(3):361. doi: 10.3390/jof9030361 (PMC10052124; doi:10.3390/jof9030361)
Supplement: Supplementary file 1 [file jof-09-00361-s001.zip › jof-2150393-supplementary_v3.pdf]

SUPPLEMENTARY INFORMATION

Exploring the mycovirus universe: Identification, Diversity, and Biotechnological Applications

Diana Carolina Villan Larios[1]; Brayan Maudiel Diaz Reyes[1]; Carlos Priminho Pirovani[1]; Leandro Lopes Loguercio[1]; Vinícius Castro Santos[2]; Aristóteles Góes-Neto[4]; Paula Luize Camargos Fonseca[3,*], Eric Roberto Guimarães Rocha Aguiar[1,*]

[1] Department of Biological Sciences, Universidade Estadual de Santa Cruz, Ilhéus, Bahia, CEP 45662900, Brazil; carolarios.312@gmail.com; 1994bmaudiel@gmail.com; pirovanicp@gmail.com; leandro@uesc.br; ericgdp@gmail.com;

[2] Department of Biochemistry and Immunology, Instituto de Ciências Biológicas, Universidade Federal de Minas Gerais, Belo Horizonte, Minas Gerais, CEP 30270-901, Brazil; vini8cs@gmail.com
[3] Department of Genetics, Instituto de Ciências Biológicas, Universidade Federal de Minas Gerais, Belo Horizonte, Minas Gerais, CEP 30270-901, Brazil; camargos.paulaluize@gmail.com
[4] Department of Microbiology, Instituto de Ciências Biológicas, Universidade Federal de Minas Gerais, Belo Horizonte, Minas Gerais, CEP 30270-901, Brazil; arigoesneto@gmail.com

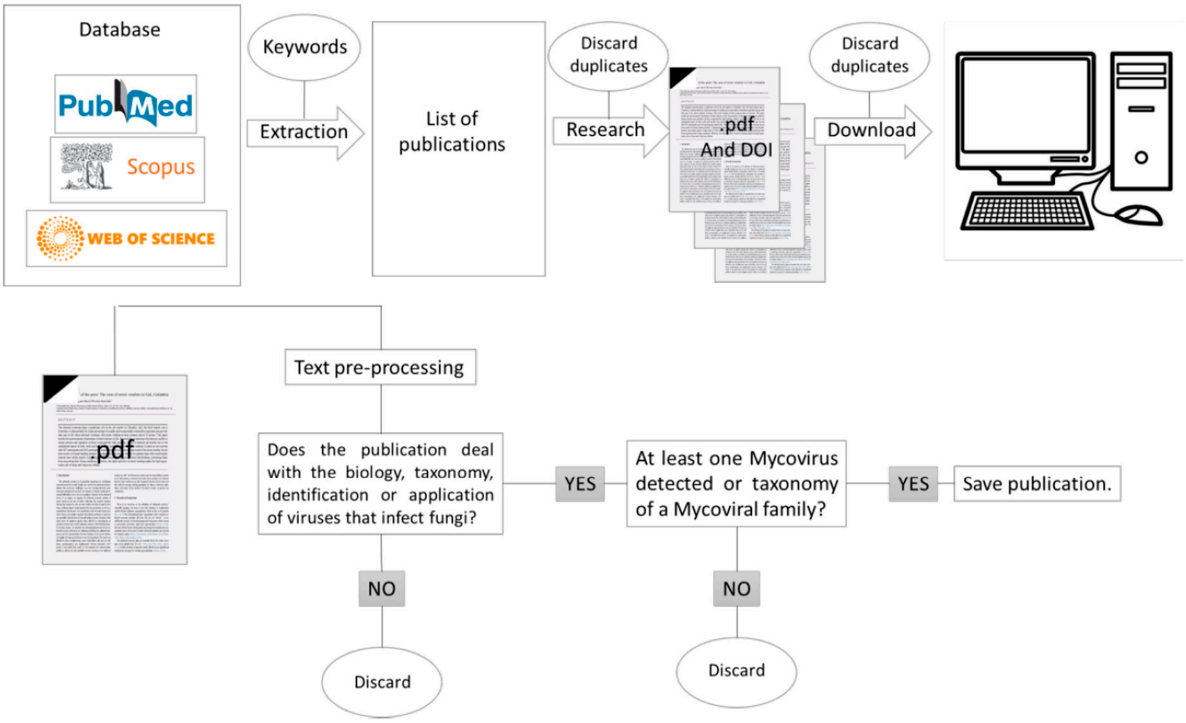

Supplementary Figure S1. Workflow for data acquisition. The databases with keywords (mycovirus and fungal viruses) were consulted to extract a list of publications, in which title and abstract were considered, and duplicates discarded. The papers were classified by groups (publications dealing with the biology, taxonomy, identification, and application of mycovirus, and including at least one mycoviral family or species) those that did not meet the criteria were discarded.

Supplementary Table S1. Classification of viruses that infect fungi, based on Report of ICTV (May, 2021), ICTV on line.

Virus						Fungus			NCBI	Fungos			
**Family**	**Genus**	**Name**	**Size**	**Genome**	**Year**	**Host**	**Family**	**Genus**		**Kingdom**	**Filo**	**Class**	**Order**
**Partitiviridae**	Alphapartitivirus	*Chondrostereum purpureum cryptic virus 1*	1920 nt	dsRNA	2013	*Chondrostereum purpureum*	Meruliaceae	*Chondrostereum*	NC_038915	FUNGI	Basidiomycota	Agaricomycetes	Polyporales
		Flammulina velutipes browning virus	1915 nt	dsRNA	2013	*Flammulina Velutipes*	Physalacriaceae	*Flammulina*	AB465308	FUNGI	Basidiomycota	Agaricomycetes	Agaricales
		Helicobasidium mompa partitivirus V70	1928 nt	dsRNA	2005	*Helicobasidium mompa*	Platygloeaceae	*Helicobasi-dium*	NC_043392	FUNGI	Basidiomycota	Pucciniomicetos	Helicobasidiales
		Heterobasidion partitivirus 1, Heterobasidion partitivirus 3, Heterobasidion partitivirus 12, Heterobasidion partitivirus 13, y Heterobasidion partitivirus 15	2027 nt, 1826 nt, 1884 nt, 1873 nt, 1791 nt	dsRNA	2013, 2013, 2015, 2015 e 2015	*Heterobasidion sp., Heterobasidion ecrustosum, Heterobasidion annosum, Heterobasidion parviporum.*	Bondarzewiaceae	*Heterobasidion*	NC_038827, NC_038835, NC_038829, NC_038831, NC_038833	FUNGI	Basidiomycota	Agaricomycetes	Russulales
		Rosellinia necatrix partitivirus 2	*1985 nt*	*dsRNA*	*2013*	*rosellinia necatrix*	Xylariaceae	*Rosellinia*	*NC_020234*	FUNGI	*Ascomycota*	*Sordariomicetos*	*Xylariales*
	Betapartitivirus	*Virus atkinsonella hypoxylon*	2180 nt	*dsRNA*	*1999*	*Atkinsonella hypoxylon*	Clavivipitaceae	*Atkinsonel la*	NC_003470	FUNGI	Ascomycota	Sordariomicetos	Hypocreales
		Ceratocystis resinifera virus 1	*2207 nt*	*dsRNA*	*2009*	*Endoconidiophora resinífera*	Ceratocystidaceae	*Ceratocystis*	*NC_010755*	FUNGI	*Ascomycota*	Sordariomicetos	*Microescalas*
		Virus fusarium poae 1	2231 nt	*dsRNA*	*2002*	*Fusarium poae*	Nectriaceae	*Fusarium*	NC_030877	FUNGI	Ascomycota	Sordariomicetos	Hypocreales
		Virus pleurotus ostratus 1	*2223 nt*	*dsRNA*	*2009*	*pleurotus ostreatus*	Pleurotaceae	*Pleurotus*	NC_006960	FUNGI	*Basidiomycota*	*Agaricomicetos*	Agaricales
		Heterobasidion partivirus 2, Heterobasidion partivirus 7, Heterobasidion partivirus 8 y Heterobasidion partivirus P	2290 nt, 2297 nt, 2281 nt, 2325 nt	*dsRNA*	*2013, 2015, 2013, 2005*	*Heterobasidion sp.,*	Bondarzewiaceae	*Heterobasidion*	*NC_038839, NC_035485, NC_038841, NC_043393*	FUNGI	Basidiomycota	Agaricomycetes	Russulales
		Rosellinia necratix virus 1 -w8	*2279 nt*	*dsRNA*	*2005*	*Rosellinia necratix*	Xylariaceae	*Rosellinia*	NC_007538	FUNGI	*Ascomycota*	*Sordariomicetos*	*Xylariales*
		Rhizoctonia solani virus 717	*2206 nt*	*dsRNA*	*1991*	*Rhizoctonia solani*	Agonomicetaceae	*Rhizoctonia*	*NC_003802*	FUNGI	*Basidiomycota*	*Agaricomicetos*	*Cantharellales*
	Gammapartitivirus	*Virus de aspergillus ochraceus*	*1220 nt*	*dsRNA*	*1991*	*Aspergillus ochreceus*	Trichocomaceae	*Aspergillus*	*NC_043395*	FUNGI	*Ascomycota*	Eurotiomycetes	Eurotiales
		Virus discula destructiva 1 y Virus discula destructiva 2	*1787 nt*	*dsRNA*	*2005, 2005*	*Discula destructiva*	Valseaceae	*Discula*	NC_002797	FUNGI	*Ascomycota*	*Sordariomicetos*	*Diaporthales*
		Fusarium solani virus 1	*1645 nt*	*dsRNA*	*2002*	*Fusarium solani, haematococca, fusarium silvicola*	Nectriaceae	*Fusarium*	NC_003885	FUNGI	Ascomycota	Sordariomicetos	*Hypocreales*

		Virus de ARN de Gremmeniella abietina MS1	*1782 nt*	*dsRNA*	*2005*	*Gremmeniella abietina*	Helotiaceae	*Gremmeniella*	NC_004018	FUNGI	*Ascomycota*	Sordariomicetos	*Helotiales*
		Ophiostoma partitivirus 1	*1567 nt*	*dsRNA*	*2009*	*Ophiostoma sp.*	Ophiostomataceae	*Ophiostoma*	NC_038917	FUNGI	*Ascomycota*	Sordariomicetos	Ophiostomatales
		Penicillium stoloniferum virus F y Penicillium stoloniferum virus S	*1677 nt, 1754 nt*	*dsRNA*	*2009, 1979*	*Penicillium stoloniferum, penicillium brevicompactum, aspergillus ochraceus*	Trichocomaceae	*Penicillium*	*NC_007221, NC_005976*	FUNGI	*Ascomycota*	Eurotiomycetes	*Eurotiales*
**Chrysoviridae**	Alphachrysovirus	*Aspergillus fumigatus chrysovirus*	*3006 nt*	*dsRNA*	*2012*	*Aspergillus fumigatus*	Trichocomaceae	*Aspergillus*	*NC_038871*	FUNGI	*Ascomycota*	Eurotiomycetes	Eurotiales
		Colletotrichum gloeosporioides chrysovirus	*3397 nt*	*dsRNA*	*2016*	*Colletotrichum gloeosporioides*	Glomerellaceae	*Colletotrichum*	NC_043666	FUNGI	*Ascomycota*	*Sordariomicetos*	*Glomerellales*
		Chrysothrix chrysovirus 1	*3552 nt*	*dsRNA*	*2020*	*Penicillium citreosulfuratum*	Trichocomaceae	*Penicillium*	MN625832	FUNGI	*Ascomycota*	*Eurotiomycetes*	*Eurotiales*
		Cryphonectria nitschkei chrysovirus 1	*2978 nt*	*dsRNA*	*2006*	*Cryphonectria nitschkei*	Cryphonectriaceae	*Cryphonectria*	*NC_038778*	FUNGI	*Ascomycota*	*Sordariomicetos*	*Diaporthales*
		Fusarium oxysporum chrysovirus 1	*2574 nt*	*SsRNA*	*2007*	*Fusarium oxysporum*	Nectriaceae	*Fusarium*	*NC_043218*	FUNGI	*Ascomycota*	*Sordariomicetos*	*Hypocreales*
		Virus Helminthosporium victoriae 145S	*3612 nt*	*RNA*	*2002*	*Helminthosporium victoriae*	Pleosporaceae	*Helminthospori*	*NC_005978*	FUNGI	Ascomycota	*Dothideomycetes*	*Pleosporales*
		Isaria Javanica Chrysovirus	*3593 nt*	*dsRNA*	*2017*	*Isara Javanica, Cordyceps javanica*	Cordycipitaceae	*Isara*	*NC_033277*	FUNGI	*Ascomycota*	*Sordariomycetes*	*Hypocreales*
		Macrophomina phaseolina chrysovirus	*3712 nt*	*dsRNA*	*2015*	*Macrophomina phaseolina*	Botryosphaeriaceae	*Macrophomina*	*NC_043662*	FUNGI	*Ascomycota*	*Dothideomycetes*	*Botryosphaeriales*
		Virus penicillium brevicompactum	*3149 nt*	*SsRNA*	*2002*	*Penicillium brevicompactum*	Trichocomaceae	*Penicillium*	MG887748	FUNGI	*Ascomycota*	*Eurotiomycetes*	*Eurotiales*
		Virus penicillium chrysogenum	*3562 nt*	*dsRNA*	*1979*	*Penicillium chrysogenum*	Trichocomaceae	*Penicillium*	*NC_007539*	FUNGI	*Ascomycota*	*Eurotiomycetes*	*Eurotiales*
		Penicillium cyaneofulvum virus		*dsRNA*	*1979*	*Penicillium cyaneofulvum*	Trichocomaceae	*Penicillium*	Sem entrada no Genbank	FUNGI	*Ascomycota*	*Eurotiomycetes*	*Eurotiales*
		Verticillium dahliae chrysovirus 1	*3313 nt*	*dsRNA*	*2010*	*Verticillium Dahliae*	Incertae	*Verticillium*	NC_038782	FUNGI	*Ascomycota*	*Sordariomicetos*	*Glomerellales*
	Betachrysovirus	*Alternaria alternata chrysovirus*	*2785 nt*	*dsRNA*	*2018*	*alternaria alternata*	Pleosporaceae	*Alternaria*	*NC_040736*	FUNGI	*Ascomycota*	*Dothideomycetes*	*Pleosporales*
		Botryosphaeria dothidea chrysovirus	*2397 nt*	*dsRNA*	*2014*	*Botryosphaeria dothidea*	Botryosphaeriaceae	*Botryosphaeria*	*NC_033476*	FUNGI	*Ascomycota*	*Dothideomycetes*	*Botryosphaeriales*
		Aspergillus thermomutatus chrysovirus 1	*3589 nt*	*dsRNA*	*2018*	*Aspergillus thermomutatus*	Trichocomaceae	*Aspergillus*	*MF045841*	FUNGI	*Ascomycota*	*Eurotiomycetes*	*Eurotiales*
		Colletotrichum fructicola chrysovirus 1	*3620 nt*	*dsRNA*	*2018*	*Colletotrichum fructicola*	glomerellaceae	*Colletotrichum*	*NC_040485*	FUNGI	Ascomycota	*Sordariomicetos*	*Glomerellales*

		Coniothyrium diplodiella chrysovirus 1	*3625 nt*	*dsRNA*	*2020*	*Coniella diplodiella*	Melanconidaceae	*Coniella*	[*MK584812*]	FUNGI	Ascomycota	*Sordariomicetos*	*Diaporthales*
		Fuarium graminearum chrysovirus	*3580 nt*	*dsRNA*	*2018*	*Fusarium graminearum*	Nectriaceae	*Fusarium*	HQ343295	FUNGI	*Ascomycota*	*Sordariomicetos*	*Hypocreales*
		Fuarium oxysporum chrysovirus 2	*3555 nt*	*dsRNA*	*2015*	*Fuarium oxysporum*	Nectriaceae	*Fusarium*	*NC_027563*	FUNGI	*Ascomycota*	*Sordariomicetos*	*Hypocreales*
		Penicillium janczewskii chrysovirus 1	*3698 nt*	*dsRNA*	*2015*	*Penicillium janczewskii*	Trichocomaceae	*Penicillium*	NC_028495	FUNGI	*Ascomycota*	*Eurotiomycetes*	*Eurotiales*
		Penicillium janczewskii chrysovirus 2	*2699 nt*	*ssRNA*	*2015*	*Penicillium janczewskii*	Trichocomaceae	*Penicillium*	*NC_043669*	FUNGI	*Ascomycota*	*Eurotiomycetes*	*Eurotiales*
		Magnaporthe oryzae chrysovirus	*2879 nt*	*dsRNA*	*2010*	*Magnaporthe oryzae, Pyricularia oryzae*	Magnaportheaceae	*Magnaporthe*	NC_038011	FUNGI	*Ascomycota*	Sordariomicetos	*Magnaporthales*
		Neofusicoccum parvum chrysovirus 1	*3698 nt*	*dsRNA*	*2020*	*Neofusicoccum parvum*	Botryosphaeriaceae	*Neodusicoccum*	NC_028495	FUNGI	*Ascomycota*	*Saccharomyceta*	*Botryosphaeriales*
**Amalgaviridae**	Zybavirus	*Zygosaccharomyces bailii virus Z*	*3157 nt*	*dsRNA*	*2018*	*Zygosaccharomyces bailii*	Saccharomycetaceae	*Zygosaccharomyces*	NC_003874	FUNGI	*Ascomycota*	*Saccharomyceta*	*Saccharomycetales*
**Curvulaviridae**	*Orthocurvulavirus*	*Curvularia orthocurvulavirus 1*		*dsRNA*	*2020*	*Curvularia sp.*	Pleosporaceae	*Curvularia*	**	FUNGI	*Ascomycota*	*Saccharomyceta*	*Pleosporales*
		Fusarium graminearum orthocurvulavirus		*dsRNA*	*2020*	*Fusarium graminearum*	Nectriaceae	*Fusarium*	**	FUNGI	*Ascomycota*	*Saccharomyceta*	*Hypocreales*
		Heterobasidion orthocurvulavirus		*dsRNA*	*2020*	*Heterobasidion sp.*	Bondarzewiaceae	*Heterobasidion*	**	FUNGI	*Basidiomycota*	*Agaricomicetos*	*Russulales*
		Lactarius rufus orthocurvulavirus 1		*dsRNA*	*2020*	*Lactarius rufus*	Russulaceae	*Lactarius*	**	FUNGI	*Basidiomycota*	*Agaricomicetos*	*Russulales*
		Rhizoctonia solani orthocurvulavirus 1		*dsRNA*	*2020*	*Rhizoctonia solani*	Ceratobasidiaceae	*Rhizoctonia*	**	FUNGI	*Basidiomycota*	*Agaricomicetos*	*Cantharellales*
		Lactarius tabidus orthocurvulavirus 1		*dsRNA*	*2020*	*Lactarius tabidus*	Russulaceae	*Lactarius*	**	FUNGI	*Basidiomycota*	*Agaricomicetos*	*Russulales*
		Sclerotium hydrophilum orthocurvulavirus 1		*dsRNA*	*2020*	*Sclerotium hydrophilum*	Typhulaceae	*Sclerotium*	**	FUNGI	*Basidiomycota*	*Agaricomicetos*	*Thelephorales*
		Trichoderma harzianum orthocurvulavirus 1		*dsRNA*	*2020*	*Trichoderma harzianum*	Hypocreaceae	*Trichoderma*	**	FUNGI	*Ascomycota*	*Sordariomicetos*	*Hypocreales*
**Hypoviridae**	*Not assigned*	*Sclerotinia sclerotiorum hypovirus 7*	*10208 nt*	*ssRNA*	*2021*	*Sclerotinia sclerotiorum*	Sclerotiniaceae	*Sclerotinia*	*MT646394*	FUNGI	*Ascomycota*	*Leotiomicetos*	*Helotiales*

		Sclerotinia sclerotiorum hypovirus 2-WX	*15229 nt*	*ssRNA*	*2021*	*Sclerotinia sclerotiorum*	Sclerotiniaceae	*Sclerotinia*	[MT646366](MT646366)	FUNGI	*Ascomycota*	*Leotiomicetos*	*Helotiales*
		Sclerotinia sclerotiorum hypovirus 8-WX1	*14659 nt*	*ssRNA*	*2021*	*Sclerotinia sclerotiorum*	Sclerotiniaceae	*Sclerotinia*	[MT646367](MT646367)	FUNGI	*Ascomycota*	*Leotiomicetos*	*Helotiales*
		Sclerotinia sclerotiorum hypovirus 8 - WX2	*14158 nt*	*ssRNA*	*2021*	*Sclerotinia sclerotiorum*	Sclerotiniaceae	*Sclerotinia*	[MT646368](MT646368)	FUNGI	*Ascomycota*	*Leotiomicetos*	*Helotiales*
		Sclerotnia sclerotiorum hypovirus 1	*10430 nt*	*ssRNA*	*2021*	*Sclerotinia sclerotiorum*	Sclerotiniaceae	*Sclerotinia*	[MT829322](MT829322)	FUNGI	*Ascomycota*	*Leotiomicetos*	*Helotiales*
	Hipovirus	*Cryphonectria hipovirus 1, Cryphonectria hipovirus 2, Cryphonectria hipovirus 3 y Cryphonectria hipovirus 4*	*12734 nt, 12507 nt, 9799 nt, 9149 nt*	*dsRNA*	*1993, 1995, 1999, 2002*	*Cryphonectria nitschkei, Cryphonectria SP., Cryphonectria parasitica* .	Cryphonectriaceae	*Cryphonectria*	[NC_001492, NC_003534, NC_000960, NC_006431](NC_001492)	FUNGI	*Ascomycota*	*Sordariomicetos*	*Diaporthales*
**Reoviridae**	Mycoreovirus	*mycoreovirus 1, mycoreovirus 2 y mycoreovirus 3*	*2269 nt, 3213 nt, 3773 nt*	*dsRNA*	*2003, 2005, 2002*	*Sordariomycetous Rosellinia necatrix fitopatógenos - Diaporthe sp., Cryphonectria parasitica , Valsa ceratosperma, Sclerotina sclerotiorum y Glomerella cingulata*	Xylariaceae , Cryphonectriaceae	*Cryphonectria*	[NC_010746, NC_038657, NC_007533](NC_010746)	FUNGI	*Ascomycota*	*Sordariomicetos*	*Diaporthales*
**Totiviridae**	Totivirus	*Virus Saccharomyces cerevisiae L-A*	*4579 nt*	*dsRNA*	*1982*	*Saccharomyces*	Saccharomycetaceae	*Saccharomyces*	[NC_003745](NC_003745)	FUNGI	*Ascomycota*	*Sordariomicetos*	*Saccharomycetales*
		Virus Saccharomyces cerevisiae LBCLa	*4615 nt*	*dsRNA*	*1991*	*Saccharomyces*	Saccharomycetaceae	*Saccharomyces*	[NC_001641](NC_001641)	FUNGI	*Ascomycota*	*Sordariomicetos*	*Saccharomycetales*
		Scheffersomyces segobiensis virus L	*4590 nt*	*dsRNA*	*2013*	*Scheffersomyces segobiensis*	Saccharomycetaceae	*Scheffersomyc*	[NC_038697](NC_038697)	FUNGI	*Ascomycota*	*Sordariomicetos*	*Saccharomycetales*
		Ustilago maydis virus H1	*6099 nt*	*dsRNA*	*1982*	*Ustilago maydis*	Ustilaginaceae	*Ustilago*	[NC_003823](NC_003823)	FUNGI	*Basidiomycota*	*Ustilaginomycetes*	*Ustilaginales*
	Victorivirus	*Aspergillus foetidus virus lento 1*	*5194 nt*	*dsRNA*	*2013*	*Aspergillus foetidus*	Trichocomaceae	*Aspergillus*	[NC_038928](NC_038928)	FUNGI	*Ascomycota*	*Eurotiomycetes*	*Eurotiales*
		Beauveria bassiana victorivirus 1	*5228 nt*	*dsRNA*	*2011*	*Beauveria bassiana*	Clavicipitaceae	*Beauveria*	[NC_038929](NC_038929)	FUNGI	*Ascomycota*	*Sordariomicetos*	*Hypocreales*
		Chalara elegans RNA Virus 1	*5310 nt*	*dsRNA*	*2009*	*Berkeleyomyces basicola*	Ceratocystidaceae	*Berkeleyomyces*	[NC_005883](NC_005883)	FUNGI	*Ascomycota*	*Sordariomicetos*	*Hypocreales*

		Coniothyrium minitans RNA virus	4975 nt	dsRNA	2003	Coniothyrium minitans	Phaeosphaeriaceae	Coniothyrium	NC_007523	FUNGI	Ascomycota	Dothideomycetes	Pleosporales
		Epichloe festucae virus 1	5109 nt	dsRNA	2006	Epichloe festucae	Clavicipitaceae	Epichloe	NC_038930	FUNGI	Ascomycota	Sordariomicetos	Hypocreales
		Gremmeniella abietina RNA virus L1	5133 nt	dsRNA	2001	Gremmeniella abietina	Helotiaceae	Gremmeniella	NC_003876	FUNGI	Ascomycota	Leotiomicetos	Helotiales
		Helminthosporium victoriae virus 190S	5179 nt	dsRNA	1990	Bipolaris victoriae	Pleosporaceae	Bipolaris	NC_003607	FUNGI	Ascomycota	Dothideomycetes	Pleosporales
		Helicobasidium mompa totivirus 1-17	5207 nt	dsRNA	2003	Helicobasidium mompa	Platygloeaceae	Helicobasidium	NC_005074	FUNGI	Basidiomycota	Pucciniomicetos	Helicobasidiales
		Magnaporthe oryzae virus 1 y Magnaporthe oryzae virus 2	5359 nt	dsRNA	2008, 2013	Magnaporthe oryzae	Magnaporthaceae	Magnaporthe	NC_006367	FUNGI	Ascomycota	Sordariomicetos	Magnaporthales
		Rosellinia necatrix victorivirus 1	5329 nt	ssRNA	2012	Rosellinia necatrix y Cryphonectria parasitica	Xylariaceae y Cryphonectriaceae	Rosellinia	NC_021565	FUNGI	Ascomycota	Sordariomicetos	Xylariales
		Sphaeropsis sapinea RNA virus 1 y Sphaeropsis sapinea RNA virus 2	5163 nt, 5202 nt	dsRNA	1998, 1998	Diplodia sapinea	Botryosphaeriaceae	Sphaeropsis	NC_001963, NC_001964	FUNGI	Ascomycota	Dothideomycetes	Botryosphaeriales
		Tolypocladium cylindrosporum virus 1	5196 nt	dsRNA	2010	Tolypocladium cylindrosporum	Ophiocordycipitaceae	Tolypocladium	NC_014823	FUNGI	Ascomycota	Sordariomicetos	Hypocreales
Endornaviridae	Alphaendornavirus	Agaricus bisporus alphaendornavirus 1	12730 nt	dsRNA	2017	Agaricus bisporus	Agaricaceae	Agaricus	KY357509	FUNGI	Basidiomycota	Agaricomicetos	Agaricales
		Erysiphe cichoracearum alphaendornavirus	11907 nt	dsRNA	2016	Golovinomyces cichoracearum	Erysiphaceae	Erysiphe	NC_029095	FUNGI	Ascomycota	Leotiomicetos	Erysiphales
		Helicobasidium mompa alphaendornavirus 1	16614 nt	dsRNA	2006	Helicobasidium mompa	Helicobasidiaceae	Helicobasidium	NC_013447	FUNGI	Ascomycota	Dothideomycetes	Pleosporales
		Rhizoctonia cerealis alphaendornavirus 1	17486 nt	dsRNA	2016	Ceratobasidium cereale	Ceratobasidiaceae	Ceratobasidium	NC_022619	FUNGI		Basidiomycota	
		Rhizoctonia solani alphaendornavirus 2	15850 nt	dsRNA	2016	Rhizoctonia solani	Ceratobasidiaceae	Rhizoctonia	KT823701	FUNGI	Basidiomycota	Agaricomicetos	Cantharellales
	Betaendornavirus	Alternaria brassicicola betaendornavirus 1	10290 nt	RNA	2014	Alternaria brassicicola	Pleosporaceae	Alternaria	NC_026136	FUNGI	Ascomycota	Dothideomycetes	Pleosporales

		Botrytis cinerea betaendornavirus 1	*11557 nt*	*RNA*	*2016*	*Botrytis cinerea*	Sclerotinicaeae	*Botrytis*	NC_031752	FUNGI	*Ascomycota*	*Leotiomycetes*	*Helotiales*
		Rosellinia necratix betaendornavirus 1	*9639 nt*	*RNA*	*2015*	*Rosellinia necratix*	Xylariaceae	*Rosellinia*	NC_030938	FUNGI	*Ascomycota*	*Sordariomicetos*	*Xylariales*
		Gremmeniella abietina betaendornavirus 1	*10375 nt*	*dsRNA*	*2006*	*Gremmeniella abietina*	Helotiaceae	*Gremmeniella*	NC_007920	FUNGI	*Ascomycota*	Sordariomicetos	*Helotiales*
		Sclerotinia minor betaendornavirus 1	*12626 nt*	*RNA*	*2018*	*Sclerotinia minor*	Sclerotiniaceae	*Sclerotinia*	NC_040631	FUNGI	*Ascomycota*	*Leotiomicetos*	*Helotiales*
		Sclerotinia sclerotiorum betaendornavirus 1	*10513 nt*	*dsRNA*	*2013*	*Sclerotinia sclerotiorum*	Sclerotiniaceae	*Sclerotinia*	NC_023893	FUNGI	*Ascomycota*	*Leotiomicetos*	*Helotiales*
		Tuber aestivum betaendornavirus	*9760 nt*	*RNA*	*2011*	*Tuber aestivum*	Tuberaceae	*Tuber*	NC_014904	FUNGI	*Ascomycota*	*Pezizomycetes*	*Pezizales*
	Not assigned	*Sclerotinia sclerotiorum endornavirus 1-WX*	*10596 nt*	*ssRNA*	*2021*	*Sclerotinia sclerotiorum*	Sclerotiniaceae	*Sclerotinia*	MT646414	FUNGI	*Ascomycota*	*Leotiomicetos*	*Helotiales*
		Sclerotinia sclerotiorum endornavirus 8	*11543 nt*	*ssRNA*	*2021*	*Sclerotinia sclerotiorum*	Sclerotiniaceae	*Sclerotinia*	MT646356	FUNGI	*Ascomycota*	*Leotiomicetos*	*Helotiales*
		Sclerotinia sclerotiorum endornavirus 9	*13562 nt*	*ssRNA*	*2021*	*Sclerotinia sclerotiorum*	Sclerotiniaceae	*Sclerotinia*	MT646421	FUNGI	*Ascomycota*	*Leotiomicetos*	*Helotiales*
		Sclerotinia sclerotiorum endornavirus 10	*2504 nt*	*ssRNA*	*2021*	*Sclerotinia sclerotiorum*	Sclerotiniaceae	*Sclerotinia*	MT646402	FUNGI	*Ascomycota*	*Leotiomicetos*	*Helotiales*
		Sclerotinia scletotiotum endornavirus 3	*12631nt*	*ssRNA*	*2021*	*Sclerotinia sclerotiorum*	Sclerotiniaceae	*Sclerotinia*	MT706020	FUNGI	*Ascomycota*	*Leotiomicetos*	*Helotiales*
**Alphaflexiviridae**	Botrexvirus	*Botrytis virus X*	*6966 nt*	*ssRNA*	*2003*	*Botrytis cinerea*	Sclerotinicaeae	*Botrytis*	NC_005132	FUNGI	*Ascomycota*	*Leotiomycetes*	*Helotiales*
	Sclerodarnavirus	*Sclerotinia sclerotiorum debilitation-associated RNA virus*	*5470 nt*	*dsRNA*	*2003*	*Sclerotinia sclerotiorum*	Sclerotinicaeae	*Sclerotinia*	NC_007415	FUNGI	*Ascomycota*	*Leotiomicetos*	*Helotiales*
**Deltaflexiviridae**	Deltaflexivirus	*Fusarium deltaflexivirus 1*	*8246 nt*	*ssRNA*	*2016*	*Fusarium sp.*	Nectriaceae	*Fusarium*	NC_030654	FUNGI	Ascomycota	Sordariomicetos	Hypocreales
		Sclerotinia deltaflexivirus 1	*8178 nt*	*ssRNA*	*2016*	*Sclerotinia sp.*	Sclerotinicaeae	*Sclerotinia*	NC_038977	FUNGI	*Ascomycota*	*Leotiomicetos*	*Helotiales*

	Not assigned	*Sclerotinia sclerotiorum deltaflexivirus 3 S1*	*7403 nt*	*ssRNA*	*2021*	*Sclerotinia sclerotiorum*	Sclerotiniaceae	*Sclerotinia*	MT706024	FUNGI	*Ascomycota*	*Leotiomicetos*	*Helotiales*
		Sclerotinia sclerotiorum deltaflexivirus 3 S2	*2560 nt*	*ssRNA*	*2021*	*Sclerotinia sclerotiorum*	Sclerotiniaceae	*Sclerotinia*	MW452524	FUNGI	*Ascomycota*	*Leotiomicetos*	*Helotiales*
		Sclerotinia sclerotiorum deltaflexivirus 2-WX	*6651 nt*	*ssRNA*	*2021*	*Sclerotinia sclerotiorum*	Sclerotiniaceae	*Sclerotinia*	MT646426	FUNGI	*Ascomycota*	*Leotiomicetos*	*Helotiales*
		Sclerotinia sclerotiorum deltaflexivirus 3	*7375 nt*	*ssRNA*	*2021*	*Sclerotinia sclerotiorum*	Sclerotiniaceae	*Sclerotinia*	MT646428	FUNGI	*Ascomycota*	*Leotiomicetos*	*Helotiales*
		Sclerotinia sclerotiorum deltaflexivirus 1-WX	*8124 nt*	*ssRNA*	*2021*	*Sclerotinia sclerotiorum*	Sclerotiniaceae	*Sclerotinia*	MT646429	FUNGI	*Ascomycota*	*Leotiomicetos*	*Helotiales*
		Sclerotinia sclerotiorum deltaflexivirus 3 S3	*1827 nt*	*ssRNA*	*2021*	*Sclerotinia sclerotiorum*	Sclerotiniaceae	*Sclerotinia*	MW452525	FUNGI	*Ascomycota*	*Leotiomicetos*	*Helotiales*
**Barnaviridae**	Barnavirus	*Mushroom bacilliform virus*	*4009 nt*	*ssRNA*	*1993*	*Agaricus bisporus*	Agaricaceae	*Agaricus*	NC_001633	FUNGI	*Basidiomycota*	*Agaricomicetos*	*Agaricales*
**Narnaviridae**	Not assigned	Contig2_gc1_gc4_2hits				*Moniliophthora roreri*	Marasmiaceae	*Moniliophthora sp*	**	FUNGI	*Basidiomycota*	*Agaricomicetos*	*Agaricales*
		Contig1_gc1_gc4_manyhits				*Moniliophthora roreri*	Marasmiaceae	*Moniliophthora sp*	**	FUNGI	*Basidiomycota*	*Agaricomicetos*	*Agaricales*
		Contig3_gc4_gc1_orfmenor_8hits				*Moniliophthora roreri*	Marasmiaceae	*Moniliophthora sp*	**	FUNGI	*Basidiomycota*	*Agaricomicetos*	*Agaricales*
		Sclerotinia sclerotiorum narnavirus 1	*2562 nt*	*ssRNA*	*2021*	*Sclerotinia sclerotiorum*	Sclerotiniaceae	*Sclerotinia*	MT646401	FUNGI	*Basidiomycota*	*Agaricomicetos*	*Agaricales*
		Sclerotinia sclerotiorum narnavirus 2	*2382 nt*	*ssRNA*	*2021*	*Sclerotinia sclerotiorum*	Sclerotiniaceae	*Sclerotinia*	MT646403	FUNGI	*Basidiomycota*	*Agaricomicetos*	*Agaricales*
		Sclerotinia sclerotiorum narnavirus 3		*ssRNA*	*2021*	*Sclerotinia sclerotiorum*	Sclerotiniaceae	*Sclerotinia*	**	FUNGI	*Basidiomycota*	*Agaricomicetos*	*Agaricales*
		Sclerotinia sclerotiorum narnavirus 4		*ssRNA*	*2021*	*Sclerotinia sclerotiorum*	Sclerotiniaceae	*Sclerotinia*	**	FUNGI	*Basidiomycota*	*Agaricomicetos*	*Agaricales*
	Narnavirus	*Narnavirus de ARN Saccharomyces 20S y Narnavirus de ARN Saccharomyces 23S*	*2514 nt , 2891 nt*	*dsRNA*	*1998, 1998*	*Saccharomyces*	Saccharomycetaceae	*Saccharomyces*	NC_004051, NC_004050	FUNGI	*Ascomycota*	*Sordariomicetos*	*Saccharomycetales*

**Pseudoviridae**	Hemivirus	*Virus Candida albicans Tca2 y Virus Candida albicans Tca5*	6980 nt, 5911 nt	*ssRNA*	*2004, 2004*	*Candida albicans*	Saccharomycetaceae	*Candida*	AF050215, AF065434	FUNGI	*Ascomycota*	*Sordariomicetos*	*Saccharomycetales*
		Virus de Saccharomyces cerevisiae Ty5		*ssRNA*	*1999*	*Saccharomyces cerevisiae*	Saccharomycetaceae	*Candida*	**	FUNGI	*Ascomycota*	*Sordariomicetos*	*Saccharomycetales*
	Pseudovirus	*Virus Saccharomyces cerevisiae Ty1, Virus de Saccharomyces cerevisiae Ty2 y Virus Saccharomyces cerevisiae Ty4*	5918 bp, 6727 bp	*ssRNA*	*1998, 1999, 1999*	*Saccharomyces cerevisiae*	Saccharomycetaceae	*Saccharomyces*	*M18706, M94164*	FUNGI	*Ascomycota*	*Sordariomicetos*	*Saccharomycetales*
**Metaviridae**	*Metavirus*	*Virus Cladosporium fulvum T-1*	*7396 nt*	*ssADN*	*1992*	*Cladosporium fulvum*	Davidiellaceae	*Cladosporium*	NC_043491	FUNGI	*Ascomycota*	*Dothideomycetes*	*Mycosphaerellales*
		Fusarium oxysporum Skippy virus	*8457 nt*	*ssADN*	*2005*	*Fusarium oxysporum*	Nectriaceae	*Fusarium*	L34658	FUNGI	*Ascomycota*	*Sordariomicetos*	*Hypocreales*
		Saccharomyces cerevisiae Ty3 virus	*5473 nt*	*ssADN*	*1998*	*Saccharomyces cerevisiae*	Saccharomycetaceae	*Saccharomyces*	M34549	FUNGI	*Ascomycota*	*Sordariomicetos*	*Saccharomycetales*
		Virus Schizosaccharomyces pombe Tf1 y Virus Schizosaccharomyces pombe Tf2	*4533 nt, 4916 nt*	*ssRNA*	*1999, 1999*	*Schizosaccharomyces pombe*	Schizosaccharomycetaceae	*Schizosaccharomyces*	*M38526, L10324*	FUNGI	*Ascomycota*	*Esquizosacaromicetos*	*Schizosaccharomycetales*
**Genomoviridae**	*Genomovirus*	*Sclerotinia sclerotiorum* hypovirulence associated DNA virus 1	*2166nt*	*ssDNA*		*Sclerotinia sclerotiorum*	Sclerotiniaceae	*Sclerotinia*	NC_013116.1	FUNGI	*Ascomycota*	*Leotiomicetos*	*Helotiales*
**Gammaflexiviridae**	*mycoflexivirus*	*Sclerotinia sclerotiorum gammaflexivirus 1*	*6818 nt*	*ssRNA*	*2021*	*Sclerotinia sclerotiorum*	Sclerotiniaceae	*Sclerotinia*	MT646392	FUNGI	*Ascomycota*	*Leotiomicetos*	*Helotiales*
		Botrytis virus F	*6827 nt*	*ssRNA*	*2000*	*Botrytis*	Sclerotinicaeae	*Botrytis*	NC_002604	FUNGI	*Ascomycota*	*Leotiomycetes*	*Helotiales*
Mymonaviridae	*Auricularimonavirus*	*Auricularimonavirus auriculariae*		*ssRNA-*	*2020*	*Auricula sp.*	Auriculaceae	*Auricula*	**	FUNGI	*Bacillariophyta*	*Bacillariophyceae*	*Surirellales*

Botrytimonavirus	*Botrytimonavirus glicinis (Dadou sclerotimonavirus)*	*6218 nt*	*ssRNA-*	*2015*	*Patogenos de raiz en cultivos de soja, Sclerotinia sclerotiorum*	Sclerotinicaeae	*Sclerotinia sp.*	KT598228	FUNGI	*Ascomycota*	*Leotiomicetos*	*Helotiales*
	Botrytimonavirus sclerotiniae		*ssRNA-*	*2018*	*Botrytis sp*	Sclerotiniaceae	*Botrytis*	**	FUNGI	*Ascomycota*	*Leotiomicetos*	*Helotiales*
	Botrytidis botrytimonavirus		*ssRNA-*	*2020*	*Botrytis sp*	Sclerotiniaceae	*Botrytis*	**	FUNGI	*Ascomycota*	*Leotiomicetos*	*Helotiales*
Penicilimonavirus	*Penicillimonavirus alphapenicillii*		*ssRNA-*	*2020*	Penicillium sp.	Trichocomaceae	penicillium	**	FUNGI	*Ascomycota*	*Eurotiomycetes*	*Eurotiales*
	Penicillimonavirus alphaplasmoparae		*ssRNA-*	*2020*	Penicillium sp.	Trichocomaceae	penicillium	**	FUNGI	*Ascomycota*	*Eurotiomycetes*	*Eurotiales*
	Penicillimonavirus betapenicillii		*ssRNA-*	*2020*	Penicillium sp.	Trichocomaceae	penicillium	**	FUNGI	*Ascomycota*	*Eurotiomycetes*	*Eurotiales*
	Penicillimonavirus deltaplasmoparae		*ssRNA-*	*2020*	Penicillium sp.	Trichocomaceae	penicillium	**	FUNGI	*Ascomycota*	*Eurotiomycetes*	*Eurotiales*
	Penicillimonavirus épsilonplasmoparae		*ssRNA-*	*2020*	Penicillium sp.	Trichocomaceae	penicillium	**	FUNGI	*Ascomycota*	*Eurotiomycetes*	*Eurotiales*
	Penicillimonavirus etaplasmoparae		*ssRNA-*	*2020*	Penicillium sp.	Trichocomaceae	penicillium	**	FUNGI	*Ascomycota*	*Eurotiomycetes*	*Eurotiales*
	Penicilimonavirus gammaplasmopara		*ssRNA-*	*2020*	Penicillium sp.	Trichocomaceae	penicillium	**	FUNGI	*Ascomycota*	*Eurotiomycetes*	*Eurotiales*
	Penicillimonavirus kilnbarnense		*ssRNA-*	*2020*	Penicillium sp.	Trichocomaceae	penicillium	**	FUNGI	*Ascomycota*	*Eurotiomycetes*	*Eurotiales*
	Penicillimonavirus zetaplasmoparae		*ssRNA-*	*2020*	Penicillium sp.	Trichocomaceae	penicillium	**	FUNGI	*Ascomycota*	*Eurotiomycetes*	*Eurotiales*
Lentimonavirus	*Lentimonavirus lentinulae*	*11563 nt*	*ssRNA-*	*2019*	*Lentinula sp.*	Marasmiaceae	*Lentinula sp*	LC466007	FUNGI	*Basidiomycota*	*Agaricomicetos*	*Agaricales*
Sclerotimonavirus	*Drop sclerotimonavirus*	*9707 nt*	*ssRNA-*	*2015*	Sclerotinia sclerotiorum	Sclerotinicaeae	*Sclerotinia sp.*	NC_043483	FUNGI	*Ascomycota*	*Leotiomicetos*	*Helotiales*

	Glycine sclerotimonavirus (Sclerotimonavirus fusarii)	*9041 nt*	*ssRNA-*	*2015*	*Patogenos de raiz en cultivos de soja, Sclerotinia sclerotiorum*	Sclerotinicaeae	*Sclerotinia sp.*	KT598225	FUNGI	*Ascomycota*	*Leotiomicetos*	*Helotiales*
	Llinois sclerotimonavirus (Sclerotimonavirus illinoisense)	*7321 nt*	*ssRNA-*	*2015*	*Sclerotinia sclerotiorum*	Sclerotinicaeae	*Sclerotinia sp.*	KT598227	FUNGI	*Ascomycota*	*Leotiomicetos*	*Helotiales*
	Sclerotina sclerotimonavirus (Sclerotimonavirus sclerotiniae)	*10002 nt*	*ssRNA-*	*2015*	*Sclerotinia sclerotiorum*	Sclerotinicaeae	*Sclerotinia sp.*	NC_025383	FUNGI	*Ascomycota*	*Leotiomicetos*	*Helotiales*
	Sclerotinia homoeocarpa TSA contig 1		*ssRNA-*	*2020*	*Sclerotinia sclerotiorum*	Sclerotinicaeae	*Sclerotinia sp.*	JW826636	FUNGI	*Ascomycota*	*Leotiomicetos*	*Helotiales*
	Sclerotimonavirus alphaplasmoparae (Mymonavirus asociado a lesión de Plasmopara viticola 1- PvaMV-1)		*ssRNA-*	*2020*	*Sclerotinia sclerotiorum*	Sclerotinicaeae	*Sclerotinia sp.*	**	FUNGI	*Ascomycota*	*Leotiomicetos*	*Helotiales*
	Esclerotimonavirus alternariae		*ssRNA-*	*2020*	*Sclerotinia sclerotiorum*	Sclerotinicaeae	*Sclerotinia sp.*	**	FUNGI	*Ascomycota*	*Leotiomicetos*	*Helotiales*
	Sclerotimonavirus betaclarireediae		*ssRNA-*	*2020*	*Sclerotinia sclerotiorum*	Sclerotinicaeae	*Sclerotinia sp.*	**	FUNGI	*Ascomycota*	*Leotiomicetos*	*Helotiales*
	Sclerotimonavirus betaplasmoparae		*ssRNA-*	*2020*	*Sclerotinia sclerotiorum*	Sclerotinicaeae	*Sclerotinia sp.*	**	FUNGI	*Ascomycota*	*Leotiomicetos*	*Helotiales*
	Botrytidis por esclerotimonavirus		*ssRNA-*	*2020*	*Sclerotinia sclerotiorum*	Sclerotinicaeae	*Sclerotinia sp.*	**	FUNGI	*Ascomycota*	*Leotiomicetos*	*Helotiales*
	Esclerotimonavirus alphaclarireediae		*ssRNA-*	*2020*	*Sclerotinia sclerotiorum*	Sclerotinicaeae	*Sclerotinia sp.*	**	FUNGI	*Ascomycota*	*Leotiomicetos*	*Helotiales*
	Sclerotimonavirus terrae		*ssRNA-*	*2020*	*Sclerotinia sclerotiorum*	Sclerotinicaeae	*Sclerotinia sp.*	**	FUNGI	*Ascomycota*	*Leotiomicetos*	*Helotiales*
Not assigned	*Sclerotinia sclerotiorum negative-stranded RNA virus 10*	*9952 nt*	*ssRNA-*	*2021*	*Sclerotinia sclerotiorum*	Sclerotiniaceae	*Sclerotinia*	MT646378	FUNGI	*Ascomycota*	*Leotiomicetos*	*Helotiales*

		Sclerotinia sclerotiorum negative-stranded RNA virus 9	*9624 nt*	*ssRNA-*	*2021*	*Sclerotinia sclerotiorum*	Sclerotiniaceae	*Sclerotinia*	MT646386	FUNGI	*Ascomycota*	*Leotiomicetos*	*Helotiales*
		Sclerotinia sclerotiorum negative-stranded RNA virus 1-WX	*9963 nt*	*ssRNA-*	*2021*	*Sclerotinia sclerotiorum*	Sclerotiniaceae	*Sclerotinia*	MT646384	FUNGI	*Ascomycota*	*Leotiomicetos*	*Helotiales*
		Sclerotinia sclerotiorum negative-stranded RNA virus 2-WX	*9623 nt*	*ssRNA-*	*2021*	*Sclerotinia sclerotiorum*	Sclerotiniaceae	*Sclerotinia*	MT646385	FUNGI	*Ascomycota*	*Leotiomicetos*	*Helotiales*
		Sclerotinia sclerotiorum negative-stranded RNA virus 11	*9982 nt*	*ssRNA-*	*2021*	*Sclerotinia sclerotiorum*	Sclerotiniaceae	*Sclerotinia*	MT646406	FUNGI	*Ascomycota*	*Leotiomicetos*	*Helotiales*
Phenuiviridae	*Lentinuvirus*	*Lentinula lentinuvirus*	*7082 nt*	*ssRNA-*	*2019*	*Lentinula sp*	Marasmiaceae	*Lentinula sp.*	LC466008	FUNGI	*Basidiomycota*	*Agaricomicetos*	*Agaricales*
Mitoviridae	*Mitovirus*	*Cryphonectria mitovirus 1*	*2728 nt*	*dsRNA*	*1994*	*Cryphonectria parasitica*	Cryphonectriaceae	*Cryphonectria*	NC_004046	FUNGI	*Ascomycota*	*Sordariomycetes*	*Diaporthales*
		Mitovirus de Ophiostoma 4, Mitovirus de Ophiostoma 5, Mitovirus de Ophiostoma 6 e Mitovirus de Ophiostoma 3a		*dsRNA*	*2002, 2002, 2002, 2002*	*Ophiostoma sp.*	Ophiostomataceae	*Ophiostoma*	**	FUNGI	*Ascomycota*	*Sordariomycetes*	*Ophiostomatales*
Polymycoviridae	*Polymycovirus*	*Aspergillus fumigatus polymycovirus 1*	*1094 nt*	*dsRNA*	*2019*	Aspergillus fumigatus	Aspergillaceae	*Aspergillus*	NC_040418	FUNGI	*Ascomycota*	*Eurotiomycetes*	*Eurotiales*
		Aspergillus spelaeus polymycovirus 1	*2384 nt*	*dsRNA*	*2019*	*Aspergillus spelaeus*	Aspergillaceae	*Aspergillus*	MG887754	FUNGI	*Ascomycota*	*Eurotiomycetes*	*Eurotiales*
		Beauveria bassiana polymycovirus 1	*2260 nt*	*dsRNA*	*2019*	*Beauveria bassiana*	Cordycipitaceae	*Beauveria*	NC_034257	FUNGI	*Ascomycota*	*Sordariomicetos*	*Hypocreales*
		Botryosphaeria dothidea virus 1	*2397 nt*	*dsRNA*	*2019*	*Botryosphaeria dothidea*	Botryosphaeriaceae	*Botryosphaeria*	NC_033476	FUNGI	*Ascomycota*	*Dothideomycetes*	*Botryosphaeriales*
		Cladosporium cladosporioides polymycovirus 1		*dsRNA*	*2019*	*Cladosporium sp.*	Cladosporiaceae	*Cladosporium*	**	FUNGI	*Ascomycota*	*Dothideomycetes*	*Cladosporiales*

		Colletotrichum camelliae polymycovirus 1		*dsRNA*	*2019*	*Colletotrichum camelliae*	Glomerellaceae	*Colletotrichum*	**	FUNGI	*Ascomycota*	*Sordariomicetos*	*Glomerellales*
		Fusarium redolens polymycovirus 1	*2470 n t*	*dsRNA*	*2019*	*Fusarium redolens*	Nectriaceae	*Fusarium*	MK609920	FUNGI	*Ascomycota*	*Sordariomicetos*	*Hypocreales*
		Magnaporthe oryzae polymycovirus 1	*2364 nt*	*dsRNA*	*2019*	*Pyricularia oryzae*	Pyriculariaceae	*Pyricularia*	MW752175	FUNGI	*Ascomycota*	*Sordariomicetos*	*Magnaporthales*
		Penicillium digitatum polymycovirus 1	*2382 nt*	*dsRNA*	*2016*	*Penicillium digitatum*	Aspergillaceae	*Penicillium*	NC_040422	FUNGI	*Ascomycota*	*Eurotiomycetes*	*Eurotiales*
		Penicillum brevicompactum polymycovirus 1	*2400 nt*	*dsRNA*	*2019*	*Penicillium brevicompactum*	Aspergillaceae	*Penicillium*	MG887750	FUNGI	*Ascomycota*	*Eurotiomycetes*	*Eurotiales*
Quadriviridae	*Quadrivirus*	*Rosellinia necatrix quadivirus 1*	*4942 nt*	*dsRNA*	*2012*	*Rosellinia necatrix*	Xylariaceae	*Rosellinia*	NC_016757	FUNGI	*Ascomycota*	*Sordariomicetos*	*Xylariales*
Megabirnaviridae	*Megabirnavirus*	*Rosellinia necratrix megabirnavirus 1*	*7180 nt*	*dsRNA*	*2009*	*Rosellinia necatrix*	Xylariaceae	*Rosellinia*	NC_013463	FUNGI	*Ascomycota*	*Sordariomicetos*	*Xylariales*
Botourmiaviridae	*Botoulivirus*	*Botrytis botoulivirus*	*2903 nt*	*ssRNA*	*2015*	*Botrytis sp., Botrytis cinerea*	Sclerotinicaeae	*Botrytis*	NC_028476	FUNGI	*Ascomycota*	*Leotiomycetes*	*Helotiales*
		Sclerotinia botoulivirus 2 y Sclerotinia botoulivirus 3	*2333 nt*	*ssRNA*	*2015, 2020*	*Sclerotinia sp.*	Sclerotiniaceae	*Sclerotinia*	KP900929	FUNGI	*Ascomycota*	*Leotiomicetos*	*Helotiales*
	Magoulivirus	*Magnaporthe magoulivirus 1*	*2364 nt*	*ssRNA*	*2016*	*Magnaporthe sp., Pyricularia oryzae*	Magnaporthaceae	*Magnaporthe*	NC_043683	FUNGI	*Ascomycota*	Sordariomicetos	*Magnaporthales*
		Cladosporium magoulivirus 1 y Cladosporium magoulivirus 2		*dsRNA*	*2019*	*Cladosporium*	Cladosporiaceae	*Cladosporium*	**	FUNGI	*Ascomycota*	*Dothideomycetes*	*Cladosporiales*
		Magnaporthe magoulivirus 1	*2364 nt*	*ssRNA*	*2018*	Pyricularia oryzae	Pyriculariaceae	*Pyricularia*	NC_043683	FUNGI	*Ascomycota*	Sordariomicetos	*Magnaporthales*
		Penicillium magoulivirus		*ssRNA*	*2020*	*Penicillium*	Aspergillaceae	*Penicillium*	**	FUNGI	*Ascomycota*	*Eurotiomycetes*	*Eurotiales*
		Phaeoacremonium magoulivirus		*ssRNA*	*2020*				**	FUNGI			
		Rhizoctonia magoulivirus 1	*2792 nt*	*ssRNA*	*2015*	*Rhizoctonia solani*	Agonomicetaceae	*Rhizoctonia*	KP900921	FUNGI	*Basidiomycota*	*Agaricomicetos*	*Cantharellales*

Penoulivirus	*Aspergillus penoulivirus*		*ssRNA*	*2020*	*Aspergillus*	Aspergillaceae		**	FUNGI			
	Cladosporium penoulivirus		*dsRNA*	*2020*	*Cladosporium*	Cladosporiaceae	*Cladosporium*	**	FUNGI	*Ascomycota*	*Dothideomycetes*	*Cladosporiales*
	Magnaporthe penoulivirus		*ssRNA*	*2020*	Pyricularia oryzae	Pyriculariaceae	*Pyricularia*	**	FUNGI	*Ascomycota*	Sordariomicetos	*Magnaporthales*
	Epicoccum penoulivirus		*ssRNA*	*2020*	*Epicoccum sp.*	Didymellaceae	*Epicoccum*	**	FUNGI	*Ascomycota*	*Dothideomycetes*	*Pleosporales*
	Neofusicoccum penoulivirus		*ssRNA*	*2020*	*Neofusicoccum sp*	Botryosphaeriaceae	*Neofusicoccum*	**	FUNGI	*Ascomycota*	*Dothideomycetes*	*Botryosphaeriales*
	Phaeoacremonium penoulivirus		*ssRNA*	*2020*	*Phaeoacremonium sp.*	Valseaceae	*Phaeoacremonium*	**	FUNGI	*Ascomycota*	*Sordariomicetos*	*Togniniales*
	Phoma penoulivirus		*ssRNA*	*2020*	*Phoma sp.*	Didymellaceae	*Phoma*	**	FUNGI	*Ascomycota*	*Dothideomycetes*	*Pleosporales*
	Phomopsis penoulivirus		*ssRNA*	*2020*	*Phomopsis*	Valsaceae	*Phomopsis*	**	FUNGI	*Ascomycota*	*Sordariomicetos*	*Diaporthales*
	Pyricularia penoulivirus		*ssRNA*	*2020*	Pyricularia oryzae	Pyriculariaceae	*Pyricularia*	**	FUNGI	*Ascomycota*	Sordariomicetos	*Magnaporthales*
	Sclerotinia penoulivirus		*ssRNA*	*2020*	*Sclerotinia sclerotiorum*	Sclerotiniaceae	*Sclerotinia*	**	FUNGI	*Ascomycota*	*Leotiomicetos*	*Helotiales*
	Penicillium penoulivirus		*ssRNA*	*2020*	*Penicillium*	Aspergillaceae	*Penicillium*	**	FUNGI	*Ascomycota*	*Eurotiomycetes*	*Eurotiales*
Rhizoulivirus	*Rhizoctonia rhizoulivirus*		*ssRNA*	*2020*	*Rhizoctonia sp.*	Ceratobasidiaceae	*Rhizoctonia*	**	FUNGI	*Basidiomycota*	*Agaricomicetos*	*Cantharellales*
Escleroulivirus	*Scleroulivirus de cladosporium*		*ssRNA*	*2020*	*Sclerotinia sclerotiorum*	Sclerotiniaceae	*Sclerotinia*	**	FUNGI	*Ascomycota*	*Leotiomicetos*	*Helotiales*
	Pyricularia scleroulivirus 2 e Pyricularia scleroulivirus 3		*ssRNA*	*2020*	Pyricularia oryzae	Pyriculariaceae	*Pyricularia*	**	FUNGI	*Ascomycota*	Sordariomicetos	*Magnaporthales*
	Sclerotinia scleroulivirus 1	*3180 nt*	*ssRNA*	*2015*	*Sclerotinia sclerotiorum*	Sclerotiniaceae	*Sclerotinia*	KP900928	FUNGI	*Ascomycota*	*Leotiomicetos*	*Helotiales*
Not assigned	*Sclerotinia sclerotiorum ourmia-like virus 5*	*2910 nt*	*ssRNA*	*2021*	*Sclerotinia sclerotiorum*	Sclerotiniaceae	*Sclerotinia*	MT646370	FUNGI	*Ascomycota*	*Leotiomicetos*	*Helotiales*
	Sclerotinia sclerotiorum ourmia-like virus 6	*2602 nt*	*ssRNA*	*2021*	*Sclerotinia sclerotiorum*	Sclerotiniaceae	*Sclerotinia*	MT646372	FUNGI	*Ascomycota*	*Leotiomicetos*	*Helotiales*

		Sclerotinia sclerotiorum ourmia-like virus 7	*3848 nt*	*ssRNA*	*2021*	*Sclerotini a sclerotior um*	Sclerotiniac eae	*Sclerotin ia*	MT6463 65	FUNGI	*Ascom ycota*	*Leotiomi cetos*	*Helotiales*
		Sclerotinia sclerotiorum ourmia-like virus 8	*2016 nt*	*ssRNA*	*2021*	*Sclerotini a sclerotior um*	Sclerotiniac eae	*Sclerotin ia*	MT6463 73	FUNGI	*Ascom ycota*	*Leotiomi cetos*	*Helotiales*
		Sclerotinia sclerotiorum ourmia-like virus 9	*1989 nt*	*ssRNA*	*2021*	*Sclerotini a sclerotior um*	Sclerotiniac eae	*Sclerotin ia*	MT6463 74	FUNGI	*Ascom ycota*	*Leotiomi cetos*	*Helotiales*
		Sclerotinia sclerotiorum ourmia-like virus 10	*2888 nt*	*ssRNA*	*2021*	*Sclerotini a sclerotior um*	Sclerotiniac eae	*Sclerotin ia*	MT6463 79	FUNGI	*Ascom ycota*	*Leotiomi cetos*	*Helotiales*
		Sclerotinia sclerotiorum ourmia-like virus 11	*2968 nt*	*ssRNA*	*2021*	*Sclerotini a sclerotior um*	Sclerotiniac eae	*Sclerotin ia*	MT6464 00	FUNGI	*Ascom ycota*	*Leotiomi cetos*	*Helotiales*
		Sclerotinia sclerotiorum ourmia-like virus 12	*2498 nt*	*ssRNA*	*2021*	*Sclerotini a sclerotior um*	Sclerotiniac eae	*Sclerotin ia*	MT6463 77	FUNGI	*Ascom ycota*	*Leotiomi cetos*	*Helotiales*
		Sclerotinia sclerotiorum ourmia-like virus 13	*2831 nt*	*ssRNA*	*2021*	*Sclerotini a sclerotior um*	Sclerotiniac eae	*Sclerotin ia*	MT6463 75	FUNGI	*Ascom ycota*	*Leotiomi cetos*	*Helotiales*
		Sclerotinia sclerotiorum ourmia-like virus 14	*3368 nt*	*ssRNA*	*2021*	*Sclerotini a sclerotior um*	Sclerotiniac eae	*Sclerotin ia*	MT6464 10	FUNGI	*Ascom ycota*	*Leotiomi cetos*	*Helotiales*
		Sclerotinia sclerotiorum ourmia-like virus 15	*3210 nt*	*ssRNA*	*2021*	*Sclerotini a sclerotior um*	Sclerotiniac eae	*Sclerotin ia*	MT6463 99	FUNGI	*Ascom ycota*	*Leotiomi cetos*	*Helotiales*
		Sclerotinia sclerotiorum ourmia-like virus 16	*2416 nt*	*ssRNA*	*2021*	*Sclerotini a sclerotior um*	Sclerotiniac eae	*Sclerotin ia*	MT6463 82	FUNGI	*Ascom ycota*	*Leotiomi cetos*	*Helotiales*
		Sclerotinia sclerotiorum ourmia-like virus 5	*2823 nt*	*ssRNA*	*2021*	*Sclerotini a sclerotior um*	Sclerotiniac eae	*Sclerotin ia*	MT7060 22	FUNGI	*Ascom ycota*	*Leotiomi cetos*	*Helotiales*
Rhabdo viridae	*Not assign ed*	*Sclerotinia sclerotiorum rhabdovirus 1*	*11356 nt*	*ssRNA -*	*2021*	*Sclerotini a sclerotior um*	Sclerotiniac eae	*Sclerotin ia*	MT7060 19	FUNGI	*Ascom ycota*	*Leotiomi cetos*	*Helotiales*
Botybirnavirus	*Not assigned*	*Sclerotinia sclerotiorum botybirnavirus 1 - SCH941*	*6457 nt*	*dsRNA*	*2015*	*Sclerotini a sclerotior um*	Sclerotiniac eae	*Sclerotin ia*	NC_0271 38	FUNGI	*Ascom ycota*	*Leotiomi cetos*	*Helotiales*
		Sclerotinia sclerotiorum botybirnavirus 2 - AH16	*6159 nt*	*dsRNA*	*2016*	*Sclerotini a sclerotior um*	Sclerotiniac eae	*Sclerotin ia*	KT96297 2	FUNGI	*Ascom ycota*	*Leotiomi cetos*	*Helotiales*
		Sclerotinia sclerotiorum botybirnavirus 3-SZ150	*6212 nt*	*dsRNA*	*2018*	*Sclerotini a sclerotior um*	Sclerotiniac eae	*Sclerotin ia*	MK5307 03	FUNGI	*Ascom ycota*	*Leotiomi cetos*	*Helotiales*

		Sclerotinia sclerotiorum botybirnavirus 1-WX	*6469 nt*	*dsRNA*	*2021*	*Sclerotinia sclerotiorum*	Sclerotiniaceae	*Sclerotinia*	MT646397	FUNGI	*Ascomycota*	*Leotiomicetos*	*Helotiales*
		Sclerotinia sclerotiorum botybirnavirus 2-WX	*6119 nt*	*dsRNA*	*2021*	*Sclerotinia sclerotiorum*	Sclerotiniaceae	*Sclerotinia*	MT646423	FUNGI	*Ascomycota*	*Leotiomicetos*	*Helotiales*
		Sclerotinia sclerotiorum botybirnavirus 3-WX	*6201 nt*	*dsRNA*	*2021*	*Sclerotinia sclerotiorum*	Sclerotiniaceae	*Sclerotinia*	MT646418	FUNGI	*Ascomycota*	*Leotiomicetos*	*Helotiales*
		Sclerotinia sclerotiorum botybirnavirus 4	*6414 nt*	*dsRNA*	*2021*	*Sclerotinia sclerotiorum*	Sclerotiniaceae	*Sclerotinia*	MT646360	FUNGI	*Ascomycota*	*Leotiomicetos*	*Helotiales*
		Sclerotinia sclerotiorum botybirnavirus 3 S1	*6214 nt*	*dsRNA*	*2021*	*Sclerotinia sclerotiorum*	Sclerotiniaceae	*Sclerotinia*	MT829323	FUNGI	*Ascomycota*	*Leotiomicetos*	*Helotiales*
		Sclerotinia sclerotiorum botybirnavirus 3 S2	*12631 nt*	*dsRNA*	*2021*	*Sclerotinia sclerotiorum*	Sclerotiniaceae	*Sclerotinia*	MT829324	FUNGI	*Ascomycota*	*Leotiomicetos*	*Helotiales*
Tymoviridae	*Not assigned*	*Sclerotinia sclerotiorum mycotymovirus 1-WX*	*7320 nt*	*ssRNA*	*2021*	*Sclerotinia sclerotiorum*	Sclerotiniaceae	*Sclerotinia*	MT646427	FUNGI	*Ascomycota*	*Leotiomicetos*	*Helotiales*
		Sclerotinia sclerotiorum mycotymovirus 1	*6414 nt*	*ssRNA*	*2021*	*Sclerotinia sclerotiorum*	Sclerotiniaceae	*Sclerotinia*	MK530705	FUNGI	*Ascomycota*	*Leotiomicetos*	*Helotiales*
Fusagraviridae	*Not assigned*	*Sclerotinia sclerotiorum fusagravirus 1*	*9018 nt*	*dsRNA*	*2021*	*Sclerotinia sclerotiorum*	Sclerotiniaceae	*Sclerotinia*	MT646357	FUNGI	*Ascomycota*	*Leotiomicetos*	*Helotiales*
		Sclerotinia sclerotiorum fusagravirus 2	*9001 nt*	*dsRNA*	*2021*	*Sclerotinia sclerotiorum*	Sclerotiniaceae	*Sclerotinia*	MT646358	FUNGI	*Ascomycota*	*Leotiomicetos*	*Helotiales*
		Sclerotinia sclerotiorum dsRNA mycovirus-L-WX1	*9095 nt*	*dsRNA*	*2021*	*Sclerotinia sclerotiorum*	Sclerotiniaceae	*Sclerotinia*	MT646387	FUNGI	*Ascomycota*	*Leotiomicetos*	*Helotiales*
		Sclerotinia sclerotiorum dsRNA mycovirus-L-WX2	*9097 nt*	*dsRNA*	*2021*	*Sclerotinia sclerotiorum*	Sclerotiniaceae	*Sclerotinia*	MT646415	FUNGI	*Ascomycota*	*Leotiomicetos*	*Helotiales*
Sclerobunyaviridae	*Not assigned*	*Sclerotinia sclerotiorum bunyavirus 1*		*ssRNA-*	*2021*	*Sclerotinia sclerotiorum*	Sclerotiniaceae	*Sclerotinia*	**	FUNGI	*Ascomycota*	*Leotiomicetos*	*Helotiales*
		Sclerotinia sclerotiorum bunyavirus 2	*8062 nt*	*ssRNA-*	*2021*	*Sclerotinia sclerotiorum*	Sclerotiniaceae	*Sclerotinia*	MT646359	FUNGI	*Ascomycota*	*Leotiomicetos*	*Helotiales*
		Sclerotinia sclerotiorum bunyavirus 3	*7707 nt*	*ssRNA-*	*2021*	*Sclerotinia sclerotiorum*	Sclerotiniaceae	*Sclerotinia*	MT646391	FUNGI	*Ascomycota*	*Leotiomicetos*	*Helotiales*

		Sclerotinia sclerotiorum bunyavirus 4		*ssRNA-*	*2021*	*Sclerotinia sclerotiorum*	Sclerotiniaceae	*Sclerotinia*	**	FUNGI	*Ascomycota*	*Leotiomicetos*	*Helotiales*
		Sclerotinia sclerotiorum bunyavirus 5	*6856 nt*	*ssRNA-*	*2021*	*Sclerotinia sclerotiorum*	Sclerotiniaceae	*Sclerotinia*	MT646405	FUNGI	*Ascomycota*	*Leotiomicetos*	*Helotiales*
		Sclerotinia sclerotiorum bunyavirus 6		*ssRNA-*	*2021*	*Sclerotinia sclerotiorum*	Sclerotiniaceae	*Sclerotinia*	**	FUNGI	*Ascomycota*	*Leotiomicetos*	*Helotiales*
		Sclerotinia sclerotiorum bunyavirus 7		*ssRNA-*	*2021*	*Sclerotinia sclerotiorum*	Sclerotiniaceae	*Sclerotinia*	**	FUNGI	*Ascomycota*	*Leotiomicetos*	*Helotiales*
Mycoaspiviridae	*Not assigned*	*Sclerotinia sclerotiorum ophiovirus-like virus 1*	*7817 nt*	*ssRNA-*	*2021*	*Sclerotinia sclerotiorum*	Sclerotiniaceae	*Sclerotinia*	MT646390	FUNGI	*Ascomycota*	*Leotiomicetos*	*Helotiales*
Botourmiaviridae	*Not assigned*	*Sclerotinia sclerotiorum ourmia-like virus 4*	*2905 nt*	*ssRNA*	*2021*	*Sclerotinia sclerotiorum*	Sclerotiniaceae	*Sclerotinia*	MT706021	FUNGI	*Ascomycota*	*Leotiomicetos*	*Helotiales*
Not assigned	*Not assigned*	*Alternaria longipes dsRNA Virus 1*	3415 nt	*dsRNA*	*2014*	*Alternaria longies*	Pleosporaceae	*Alternaria*	NC_024703	FUNGI	*Basidiomycota*	*Agaricomicetos*	*Cantharellales*
		ustilaginoidea Virens unassigned RNA virus HNND1	*2903 nt*	*dsRNA*	*2015*	ustilaguno idea virens	Incertae	*Ustilaginoidea*	NC_027427	FUNGI	*Ascomycota*	*Sordariomicetos*	*Hypocreales*
		Ustilaginoidea virens RNA virus M	*2714 nt*	*dsRNA*	*2014*	ustilaguno idea virens	Incertae	*Ustilaginoidea*	NC_025367	FUNGI	*Ascomycota*	*Sordariomicetos*	*Hypocreales*
		Colletotrichum higginsianum non-segmented dsRNA virus 1	*2923nt*	*dsRNA*	*2015*	Colletotrichum higginsianum	Glomerellaceae	*Colletotrichum*	NC_028242	FUNGI	*Ascomycota*	*Sordariomicetos*	*Glomerellales*
		Trichoderma harzianum bipartite mycovirus 1	*2088 nt*	*dsRNA*	*2018*	Trichoderma harzianum	Hypocreaceae	Trichoderma	NC_040768	FUNGI	*Ascomycota*	*Sordariomicetos*	*Hypocreales*
		Sclerotinia sclerotiorum mycotymovirus 1-WX	*7320 nt*	*ssRNA*	*2021*	*Sclerotinia sclerotiorum*	Sclerotiniaceae	*Sclerotinia*	MT646427	FUNGI	*Ascomycota*	*Leotiomicetos*	*Helotiales*
		Sclerotinia sclerotiorum mycoalphavirus virus 1	*7531 nt*	*ssRNA*	*2021*	*Sclerotinia sclerotiorum*	Sclerotiniaceae	*Sclerotinia*	MT706025	FUNGI	*Ascomycota*	*Leotiomicetos*	*Helotiales*
		Sclerotinia sclerotiorum virga-like virus 1	*1800 nt*	*ssRNA*	*2021*	*Sclerotinia sclerotiorum*	Sclerotiniaceae	*Sclerotinia*	MT646383	FUNGI	*Ascomycota*	*Leotiomicetos*	*Helotiales*
		Sclerotinia sclerotiorum umbra-like virus 3-WX1	*4088 nt*	*ssRNA*	*2021*	*Sclerotinia sclerotiorum*	Sclerotiniaceae	*Sclerotinia*	MT646363	FUNGI	*Ascomycota*	*Leotiomicetos*	*Helotiales*

	Sclerotinia sclerotiorum umbra-like virus 3-WX2	*3945 nt*	*ssRNA*	*2021*	*Sclerotinia sclerotiorum*	Sclerotiniaceae	*Sclerotinia*	MT646364	FUNGI	*Ascomycota*	*Leotiomicetos*	*Helotiales*
	Hubei sclerotinia RNA virus 1-WX	*4222 nt*	*ssRNA*	*2021*	*Sclerotinia sclerotiorum*	Sclerotiniaceae	*Sclerotinia*	MT646396	FUNGI	*Ascomycota*	*Leotiomicetos*	*Helotiales*
	Sclerotinia sclerotiorum RNA virus L-WX	*2746 nt*	*ssRNA*	*2021*	*Sclerotinia sclerotiorum*	Sclerotiniaceae	*Sclerotinia*	MT646376	FUNGI	*Ascomycota*	*Leotiomicetos*	*Helotiales*
	Sclerotinia sclerotiorum fusarivirus 2	*6281 nt*	*ssRNA*	*2021*	*Sclerotinia sclerotiorum*	Sclerotiniaceae	*Sclerotinia*	MT646393	FUNGI	*Ascomycota*	*Leotiomicetos*	*Helotiales*
	Penicillium miczynskii RNA virus 1	*2891 nt*	*dsRNA*	*2019*	Penicillium miczynskii	Trichocomaceae	Penicillium	MK584820	FUNGI	*Ascomycota*	*Eurotiomycetes*	*Eurotiales*
	Fusarium culmorum virus 1	*2898 nt*	*dsRNA*	*2020*	Fusarium culmorum	Nectriaceae	*Fusarium*	MN187541	FUNGI	Ascomycota	Sordariomicetos	Hypocreales

** Without access to GenBank

Supplementary Table S2. Mycoviruses genomic information: genome size, ORF prediction, conserved domains, and probable genetic code.

Family	Genome size (Kb)	Segments	Genes that encode	Reference studies	Genome type	Reference
**Partitiviridae**	3 - 4.8	2	RdRp, CP	Biocontrol<br>Vainio et al., 2017	dsRNA	Nibert et al., 2013
Chrysoviridae	8.9 - 16	4	RdRp, CP, P3, P5	Associated with hypovirulence, application not developed.<br>Ejmal et al., 2018; Jamal et al., 2010; Torres-Trenas et al., 2019	dsRNA	Kotta-Loizou & Coutts, 2017
Hypoviridae	9 - 13	1	RdRp	Associated with hypovirulence, application not developed. Choi et al., 2012	ssRNA+	Picarelli e<br>t al., 2019
Reoviridae	18,2 - 30,5	10 - 12	Vp1 pol, VP2, VP3, VP4, NSp1, VP6, NSp3, NSp2, VP7, NSp4, NSp5	Study of proteins associated with signs<br>Kanematsu et al., 2010	dsRNA	J. Wang et al., 2015
Totiviridae	4,6 - 7	1	RdRp, CP	protein study Chiba et al., 2013; Kotta-Loizou et al., 2020; Lukša et al., 2017	dsRNA	Kartali et al., 2019
Endornaviridae	14 - 17,6	1	RdRp	transmission mechanisms Revill et al., 1994	dsRNA	Rossmann et al., 2021
Alphaflexiviridae	5,5 - 9	1	RdRp, CP, TGB1, TGB3	N/A	ssRNA+	Kreuze et al., 2020
Betaflexiviridae	6,5 - 9	1	RdRp, CP, TGB1, TGB3	N/A	ssRNA+	Kreuze et al., 2020
Gammaflexiviridae	6,8	1	RdRp, CP	N/A	ssRNA+	Picarelli et al., 2019
Deltaflexiviridae	6 - 8	1	RdRp, poliproteinas	N/A	ssRNA+	Hamid et al., 2018
Barnaviridae	4	1	RdRp, CP, poliproteinas	N/A	ssRNA+	Marzano et al., 2016
Narnaviridae	2,5 - 2,9	1	RdRp	N/A	ssRNA+	Marzano et al., 2016
Pseudoviridae	--	1	--	N/A	ssRNA+	Koch et al., 2021
Metaviridae	3 - 15	1	RdRp, CP, NC	N/A	ssDNA+	Jacquat et al., 2020
Mymonaviridae	10	1	poliproteinas	transmission mechanisms Magae, 2012	ssRNA-	Picarelli et al., 2019
Phenuiviridae	1,7 - 6,4	3	N, NS, poliproteinas	N/A	ssRNA-+	Guo et al., 2021
Quadriviridae	16,8	4	P2, RdRp, P4	N/A	dsRNA	Picarelli et al., 2019

**Megabirnaviridae**	7 - 9	2	CP, RdRp	N/A	dsRNA	Sato et al., 2019
**Botourmiaviridae**	23,8	1 - 2	CP, RdRp	N/A	ssRNA+	Linnakoski et al., 2021
**Rhabdoviridae**	11 - 15	2	poliproteinas	N/A	ssRNA -	Walker et al., 2018; Mu et al., 2021
**Tymoviridae**	6 - 7,5	1	poliprotein	MA	ssRNA+	Martelli et al., 2002
Amalgaviridae	--	--	--	N/A	dsRNA	Zhu et al., 2021
Curvulaviridae	--	--	--	N/A	dsRNA	Zhu et al., 2021
Mitoviridae	--	--	--	N/A	dsRNA	Jacquat et al., 2020
**Botybirnavirus**	--	--	--	NA	dsRNA	Ruiz-Padilla et al., 2021
**Fusagraviridae**	--	--	--	NA	dsRNA	Jacquat et al., 2020
**Sclerobunyaviridae**	--	--	--	NA	ssRNA-	Sutela et al., 2020
Polymycoviridae	--	--	--	N/A	dsRNA	Zhu et al., 2021
**Mycoaspiviridae**	--	--	--	NA	ssRNA-	Rumbou et al., 2021
**Genomoviridae**	2.17	1	CP, polyprotein	NA	ssDNA	Fu et al., 2022

** RdRp – RNA-dependent RNA Polymerase; CP – Capsid Protein, NC – Nucleocapside, P3 – polymerase, P5 – promoting region, Vp1 pol, polymerase RdRp; VP2 – capsular protein; VP3 – nucleus capsid,- VP4- enzyme (guanylyl-transferase); NSp1, non-structural (associated with microtubule formation); VP6 - Helicase, NTPase NSp3 - variable proteins (associated with virulence); NSp2 - RNA binding associated with the outer capsid; VP7 – formation of the nucleus; NSp4 - transmembrane glycoprotein NSp5- microtubule formation; TGB1 – βb; TGB3 - βc (associated with the membrane); N – Nucleocapside; NS - non-structural proteins

Supplementary Table S3. Information about phytopathogenic fungal species described to be infected by viruses and their hosts.

Fungal Family	Fungal specie	Plant	Author
Meruliaceae	*Chondrostereum purpureum*	Rose	Francia., et al 2017
Meruliaceae	*Chondrostereum purpureum*	Plum	Bus., et al 1998
Meruliaceae	*Chondrostereum purpureum*	Cherry	Børve J., et al 2017
Meruliaceae	*Chondrostereum purpureum*	Peach	Grinbergs., et all 2021
Meruliaceae	*Chondrostereum purpureum*	Apple tree	Ozgonen H., et al 2006
Meruliaceae	*Chondrostereum purpureum*	Almond	Grinbergs., et all 2020
Platygloeaceae	*Helicobasidium mompa*	Potato	Hong., et al 2011
Bondarzewiaceae	*Heterobasidion sp., Heterobasidion ecrustosum, Heterobasidion annosum, Heterobasidion parviporum.*	Conifers	Yuan., et al 2021
Bondarzewiaceae	*Heterobasidion sp., Heterobasidion ecrustosum, Heterobasidion annosum, Heterobasidion parviporum.*	Firs	Yuan., et al 2022
Xylariaceae	*rosellinia necatrix*	Lenhosous species	Pliego., et al 2012
Xylariaceae	*rosellinia necatrix*	Semi-woody species	Pliego., et al 2012
Xylariaceae	*rosellinia necatrix*	Ornamental species	Pliego., et al 2012
Clavivipitaceae	*Atkinsonella hypoxylon*	Pastures	Clay K. 1984
Clavivipitaceae	*Atkinsonella hypoxylon*	Junco	Clay K. 1984
Ceratocystidaceae	*Endoconidiophora resinifera*	Conifers	Zubaer., et al 2018
Nectriaceae	*Fusarium poae*	Cereal	Gil-Serna., et al 2014
Agonomicetaceae	*Rhizoctonia solani*	Cereal	Wallon., et al 2020
Agonomicetaceae	*Rhizoctonia solani*	Vegetables	Kuramae., et al 2003
Agonomicetaceae	*Rhizoctonia solani*	Coffee	Kuramae., et al 2004
Valseaceae	*Discula destructiva*	Dogwood trees	Mantooth et al, 2017
Nectriaceae	*Fusarium solani, haematococca, fusarium silvicola*	Conifers	Šišić et al, 2018
Helotiaceae	*Gremmeniella abietina*	Conifers	Botella and hantula, 2018
Trichocomaceae	*Penicillium stoloniferum, penicillium brevicompactum, aspergillus ochraceus*	Apples	Detroy and still, 1974
Trichocomaceae	*Penicillium stoloniferum, penicillium brevicompactum, aspergillus ochraceus , Aspergillus fumigatus*	Mushrooms	Detroy and still, 1974
Trichocomaceae	*Penicillium stoloniferum, penicillium brevicompactum, aspergillus ochraceus , Aspergillus fumigatus*	Ginger	Detroy and still, 1974

Fungal Family	Fungal specie	Plant	Author
Trichocomaceae	*Penicillium stoloniferum, penicillium brevicompactum, aspergillus ochraceus , Aspergillus fumigatus*	Avocado	Detroy and still, 1974
Glomerellaceae	*Colletotrichum gloeosporioides*	Quercus	Choub et al 2021
Glomerellaceae	*Colletotrichum gloeosporioides*	Cassava	Choub et al 2021
Glomerellaceae	*Colletotrichum gloeosporioides*	Peach	Liang et al 2021
Glomerellaceae	*Colletotrichum gloeosporioides*	Pear	Wang et al 2021
Glomerellaceae	*Colletotrichum gloeosporioides*	Auchuba	Liang et al 2021
Glomerellaceae	*Colletotrichum gloeosporioides*	Papaya	Wang et al 2021
Glomerellaceae	*Colletotrichum gloeosporioides*	Melon	Liang et al 2021
Glomerellaceae	*Colletotrichum gloeosporioides*	Tobacco	Choub et al 2021
Cryphonectriaceae	*Cryphonectria nitschkei*	Bulbs	shahi et al 2020
Cryphonectriaceae	*Cryphonectria nitschkei*	Tomato	shahi et al 2020
Cryphonectriaceae	*Cryphonectria nitschkei*	Carnation	shahi et al 2020
Cryphonectriaceae	*Cryphonectria nitschkei*	Chrysanthemums	shahi et al 2020
Nectriaceae	*Fusarium oxysporum*	Wheat	Jangir et al 2021
Nectriaceae	*Fusarium oxysporum*	Oatmeal	Jangir et al 2021
Nectriaceae	*Fusarium oxysporum*	Corn	Jangir et al 2021
Pleosporaceae	*Helminthosporium victoriae*	Cereal	Soldevila and Ghabrial, 2000
Pleosporaceae	*Helminthosporium victoriae*	Tomato	Li et al 2011
Pleosporaceae	*Helminthosporium victoriae*	Wheat	Huang and Ghabrial, 1996
Pleosporaceae	*Helminthosporium victoriae*	Papaya	Soldevila and Ghabrial, 2000
Botryosphaeriaceae	*Macrophomina phaseolina*	, SesameMani, Cabbage, Pepper, Chickpea, Soy, Sunflower, Sweet Potato, Alfalfa, Potato, Tomato	Marquez et al 2021
Incertae	*Verticillium Dahliae, Fusarium graminearum, Fuarium oxysporum*	Sunflower	Martin-Sanz et al 2018
Incertae	*Verticillium Dahliae, Fusarium graminearum, Fuarium oxysporum*	Cauliflower	Bhat and Subbarao, 1999
Incertae	*Verticillium Dahliae, Fusarium graminearum, Fuarium oxysporum*	Cotton	Li et al 2019
Incertae	*Verticillium Dahliae, Fusarium graminearum, Fuarium oxysporum*	Lettuce	Woolliams, 1966
Incertae	*Verticillium Dahliae, Fusarium graminearum, Fuarium oxysporum*	Olive	Jiménez-Ruiz et al 2019

Fungal Family	Fungal specie	Plant	Author
Incertae	*Verticillium Dahliae, Fusarium graminearum, Fuarium oxysporum*	Potato	Song et al 2020
Incertae	*Verticillium Dahliae, Fusarium graminearum, Fuarium oxysporum*	Tobacco	Song et al 2020
Incertae	*Verticillium Dahliae, Fusarium graminearum, Fuarium oxysporum*	Tomato	Acharya et al 2020
Melanconidaceae	*Coniella diplodiella*	Grape	Liu et al 2020
Pleosporaceae	*alternaría alternata*	Tomato, Wheat, Papaya	López Couso et al 2021
Magnaporthaceae	*Magnaporthe oryzae*	Rice	Sella et al 2021
Botryosphaeriaceae	*Neofusicoccum parvum*	Grape, Mango, Olive, Eucalyptus, Maple, Oak	Leal et al 2021
Typhulaceae	*Sclerotium hydrophilum*	Rice	Gutiérrez, 2007
Sclerotiniaceae	*Sclerotinia sclerotiorum*	Potato, Beans, Lettuce, Tomato, Celery, Cauliflower, Cabbage, Carrot, Grape	Xia et al 2020
Cryphonectriaceae	*Cryphonectria parasitica*	Quercus	Rigling and Prospero, 2018
Ustilaginaceae	*Ustilago maydis*	Corn	Kretschmer et al 2017
Ceratocystidaceae	*Berkeleyomyces basicola*	Alfalfa	Gonçalves Silva et al 2021
Ceratocystidaceae	*Berkeleyomyces basicola*	African daisy, Carrot, Chickpea, Citrus, Catton, Curcubetaceae, Line, Geranium, Lentils, Green peas, Peanut, Poinsettia, Red clover, Tobacco, Tomato, Verbena	Trojak-Goluch et al 2016
Pleosporaceae	*Bipolaris victoriae*	Cereal	García-León et al 2013
Botryosphaeriaceae	*Diplodia sapinea*	Conífers	Van der Nest et al 2014
Erysiphaceae	*Golovinomyces cichoracearum*	Melon	Delhey et al 2003
Erysiphaceae	*Golovinomyces cichoracearum*	Cucumber	Cortez, 2008
Erysiphaceae	*Golovinomyces cichoracearum*	Pumpkin	Delhey et al 2004
Pleosporaceae	*Alternaria brassicicola*	Chinese cabbage, Pak choi	Calmes et al 2015
Nectriaceae	*Fusarium redolens*	Wheat, Barley	Ölmez and Tunali, 2019
Marasmiaceae	*Moniliophthora sp.*	Cocoa	Barbosa et al 2018
Davidiellaceae	*Phoma sp.*	Tomato	Chen et al 2015
Davidiellaceae	*Cladosporium fulvum*	Tomato	Wit et al 2012
Botryosphaeriaceae	*Botryosphaeria dothidea*	Grape, Mango, Olive, Eucalyptus, Maple, Oak	Dong and Gou, 2020

**Fungal Family**	**Fungal specie**	**Plant**	**Author**
Glomerellaceae	*Colletotrichum camelliae*	Tea	Wang et al 2016
Botryosphaeriaceae	*Neofusicoccum sp*	Eucalyptus	Marais et al 2021
Glomerellacea	*Colletotrichum higginsianum*	Arabidopsis	Korn et al 2015
